# Cerebral Microstructure in Aging Using Advanced Quantitative MRI

**DOI:** 10.1093/geroni/igaa057.2771

**Published:** 2020-12-16

**Authors:** Mustapha Bouhrara, Nikkita Khattar, Richard Kim, Wenshu Qian, Joseph Alisch, Luigi Ferrucci, Susan Resnick, Richard Spencer

**Affiliations:** National Institute on Aging, Bethesda, Maryland, United States

## Abstract

White matter (WM) maintenance through oligodendrocyte metabolism is an energy-intensive process. Myelin homeostasis is sensitive to hypoxia, ischemia, or hypoperfusion. Besides substrate delivery, adequate cerebral blood flow (CBF) is crucial for removal of metabolic byproducts such as iron. While some data show decreased myelin content and CBF with aging, the association between CBF and myelination is unknown. Further, breakdown of oligodendrocyte and iron may potentiate myelin loss through oxidative mechanisms. Whether iron deposition is related to myelination is unclear. Using advanced MRI methodology, we investigated associations between CBF deficits, myelin loss, and iron deposition in cognitively normal individuals. We found significant association between i) CBF deficits and myelin loss (N=67,age24-88), ii) myelin loss and iron accumulation (N=92,age21-94), and iii) CBF deficits and iron accumulation (N=35,age22-88) in critical brain structures. This work may lay the foundation for further investigations of age-related WM degeneration and markers to differentiate normal from abnormal alterations.

